# Current Food Consumption amongst the Spanish ANIBES Study Population

**DOI:** 10.3390/nu11112663

**Published:** 2019-11-05

**Authors:** Teresa Partearroyo, María de Lourdes Samaniego-Vaesken, Emma Ruiz, Javier Aranceta-Bartrina, Ángel Gil, Marcela González-Gross, Rosa M. Ortega, Lluis Serra-Majem, Gregorio Varela-Moreiras

**Affiliations:** 1 Departamento de Ciencias Farmacéuticas y de la Salud, Facultad de Farmacia, Universidad San Pablo-CEU, CEU Universities, Urbanización Montepríncipe, 28925 Alcorcón, Madrid, Spain; t.partearroyo@ceu.es (T.P.); l.samaniego@ceu.es (M.d.L.S.-V.); 2 CIBERESP, Consortium for Biomedical Research in Epidemiology and Public Health, Carlos III Health Institute, 28029 Madrid, Spain; e.ruiz@externos.isciii.es; 3 National Center for Epidemiology, Carlos III Health Institute, 28029 Madrid, Spain; 4 Department of Food Sciences and Physiology, University of Navarra, Pamplona, 31009 Navarra, Spain; javieraranceta@gmail.com; 5 Research Institute of Biomedical and Health Sciences, University of Las Palmas de Gran Canaria, 35016 Las Palmas, Spain; lluis.serra@ulpgc.es; 6 Department of Physiology, Faculty of Medicine, University of the Basque Country (UPV/EHU), 48940 Leioa, Vizcaya, Spain; 7 CIBEROBN, Biomedical Research Networking Center for Physiopathology of Obesity and Nutrition, Carlos III Health Institute, 28029 Madrid, Spain; agil@ugr.es (Á.G.); marcela.gonzalez.gross@upm.es (M.G.-G.); 8 Department of Biochemistry and Molecular Biology II and Institute of Nutrition and Food Sciences, University of Granada, 18010 Granada, Spain; 9 ImFINE Research Group, Department of Health and Human Performance, Universidad Politécnica de Madrid, 28040 Madrid, Spain; 10 Department of Nutrition and Food Science, Faculty of Pharmacy, Madrid Complutense University, 28040 Madrid, Spain; rortega@ucm.es; 11 Research Institute of Biomedical and Health Sciences, University of Las Palmas de Gran Canaria, 35016 Las Palmas, Spain; 12 Service of Preventive Medicine, Complejo Hospitalario Universitario Insular Materno Infantil (CHUIMI), Canary Health Service, Las Palmas de Gran Canaria, 35016 Las Palmas, Spain; 13 Spanish Nutrition Foundation (FEN), 28010 Madrid, Spain

**Keywords:** food group, food subgroup, consumption, intake, dietary pattern, ANIBES study, Spanish population

## Abstract

Dietary habits amongst the Spanish population are currently a relevant cause for concern, as macronutrient profiles and micronutrient intakes seem to be inadequate and globally moving away from the traditional Mediterranean dietary pattern. However, recent food consumption patterns have not been fully assessed. In the present study, our aim was therefore to describe the current food consumption from the “anthropometric data, macronutrients and micronutrients intake, practice of physical activity, socioeconomic data and lifestyles in Spain” (ANIBES) study population by assessing data defined by age and gender. The ANIBES study is a cross-sectional study of a nationally representative sample of the Spanish population. A three-day dietary record was used to obtain information about food and beverage consumption. The sample comprised 2009 individuals aged 9–75 years, plus a boost sample for the youngest age groups (9–12, 13–17, and 18–24 years, *n* = 200 per age group). The most consumed food group across all age segments were non-alcoholic beverages followed by milk and dairy products and vegetables. Consumption of cereals and derivatives, milk and dairy products, sugars and sweets, and ready-to-eat meals by children was significantly higher than those by the adult and older adult populations (*p* ≤ 0.05). Conversely, intakes of vegetables, fruits, and fish and shellfish were significantly higher in adults and older adults (*p* ≤ 0.05). In order to comply with recommendations, adherence to the Mediterranean dietary patterns should be strengthened, especially amongst younger population groups. Therefore, substantial nutritional interventions may be targeted to improve the Spanish population’s dietary patterns nowadays.

## 1. Introduction

Food habits and consumption in Spain have experienced a significant change in recent years, leading to a less varied food choice and a tendency towards an increasingly “westernized” diet [1,2,3,4]. Namely, the contribution of food and beverage groups to the daily energy intake of the Spanish population from the ANIBES study show that a higher percentage is supplied by cereals and derivatives (27.4%), with meat and meat products and oils and fats being second (15.2%) and third (12.3%), respectively [5]. Therefore, the Spanish diet is characterized by a high content of proteins (derived from fatty, domesticated, and processed meats), saturated fats, refined grains, and sugars. However, the major food groups and subgroups that encompass our dietary patterns remain unassessed.

The current dietary patterns from the Spanish population are moving away from the traditional Mediterranean diet and comprise a higher intake of animal instead of vegetal products such as legumes, nuts, fruits, and vegetables. This concern has already been expressed by other authors, as a high proportion of Spanish young children and adolescents show an even greater distance from the balanced patterns from their adult and elder counterparts [6,7]; potentially, our future generations may not enjoy the health and cultural well-known benefits of the Mediterranean diet [8,9].

At present, it is widely recognized that a suboptimal diet is a major preventable risk factor for non-communicable diseases (NCDs), which in turn, are the leading cause of mortality and disability worldwide [10]. It is estimated that 11 million (95% uncertainty interval (UI), 10–12) deaths worldwide in 2017 can be attributed to dietary risk factors amongst adults aged 25 years or older [10]. The authors emphasized that intakes of specific foods, within the context of a global healthy diet, instead of macronutrients or micronutrients, might be most relevant for NCD risk [11]. Therefore, the assessment of the consumption of major food groups and subgroups becomes a relevant task.

To date, the study of macro- and micronutrient intakes from the Spanish population has been improved and updated. However, their current food patterns have not been assessed in depth. In the past, most studies analyzed data from the Household Consumption Surveys by the Spanish Ministry of Agriculture, Fisheries and Food, but these might not accurately reflect actual individual population intakes or estimate age and gender differences. Hence, in the present study, our objectives were to analyze and describe the current food patterns from the Spanish ANIBES study population. Consequently, we aimed to assess food group and subgroup consumption defined by age and gender groups of the participants in the ANIBES study as a representative sample of the Spanish population.

## 2. Materials and Methods

The complete design, protocol and methodology of the ANIBES study (“anthropometric data, macronutrients and micronutrients intake, practice of physical activity, socioeconomic data, and lifestyles in Spain”) have been described in detail elsewhere [5,12].

### 2.1. Sample

The ANIBES study is a cross-sectional study conducted using stratified multistage sampling. The fieldwork was performed at 128 sampling points across Spain. The design of the study aimed to define a sample size that was representative of all individuals living in Spain, aged 9–75 years, and living in municipalities of at least 2000 inhabitants. The initial potential sample consisted of 2634 individuals, and the final sample comprised 2009 individuals (1013 men, 50.4%; 996 women, 49.6%). In addition, for the youngest age groups (9–12, 13–17, and 18–24 years), a “boost sample” was included to provide at least *n* = 200 per age group (error ±6.9%). Therefore, the random sample plus boost comprised 2285 participants. The sample quotas according to the following variables were age groups (9–12, 13–17, 18–64, and 65–75 years) and gender (male/female). The fieldwork for the ANIBES study was conducted from mid-September 2013 to mid-November 2013, and two previous pilot studies were performed. The final protocol was approved by the Ethical Committee for Clinical Research of the Region of Madrid, Spain. The study was coded as “FEN 2013” and approved on 31 May 2013 [5].

### 2.2. Food and Beverage Records and Adequacy of Intakes

Study participants were provided with a tablet device (Samsung Galaxy Tab 2 7.0, Samsung Electronics, Suwon, Korea) and trained in recording information by taking photos of all food and drinks consumed during the 3 days of the study, both at and outside the home. Different days of the week would be equally represented as each 3 day cycle included two working days and one weekend day. Photos had to be taken before and after each eating and drinking occasion, in order to record the actual intake. Additionally, a brief description of the meals, recipes, food brands, and other relevant information were recorded using the tablet. Participants who declared that they were unable to use the device were offered other options, such as using a digital camera, handwritten records, and/or telephone interviews. A total of 79% of the sample used a tablet, 12% a digital camera, and 9% opted for a telephone interview. Participants provided detailed information regarding each eating and drinking occasion including not only what and how much was eaten, but also where and with whom they shared their meals, and if they were watching television and/or sitting at a table. In addition, they were asked to declare if their intake was representative for that specific day (e.g., in case of a medical condition such as gastroenteritis that might compromise dietary habits) and to record any dietary supplement taken. The survey also included a series of questions about participants’ customary eating habits (e.g., the type of milk usually consumed) to facilitate further coding. Food records were returned from the field in real time to be coded by trained personal who were supervised by dieticians.

### 2.3. Statistical Analysis

Results are expressed as means ± standard deviation (SD) per group. Comparisons among groups were performed using a Student’s *t*-test for independent samples to evaluate differences by sex within the whole population and within each age group. One-way analysis of variance (ANOVA) tests with Bonferroni correction for multiple comparisons was used to calculate differences among each age group. Differences were considered significant at *p* < 0.05. Data analysis was performed with the SPSS 24.0 software package (IBM Corp., Armonk, NY, USA).

## 3. Results

The statistical description of the ANIBES population sample is included in Table 1. Data analysis on food consumption allows estimation of the average Spanish daily menu and the associated distribution of the different food groups, as shown in Table 2. Non-alcoholic beverage consumption was quantitatively the most important in the present Spanish diet followed by milk and dairy products, vegetables, fruits, grains, and meat and meat products. The most relevant consumption, within the group of non-alcoholic beverages, was associated with bottled water followed by sugary soft drinks (Table 2), whereas within the milk and dairy products, the highest consumption was recorded for liquid milk. In addition, we observed that men consumed significantly higher quantities of cereals and derivatives, meat and meat products, eggs, ready-to eat meals, sauces and condiments, and alcoholic beverages than women. However, females had higher intakes of fruits (*p* < 0.05) when compared to males (Table 2).

Similarly, in all age segments of the ANIBES population, the most consumed food group was non-alcoholic beverages (mainly water) followed by milk and dairy products and vegetables. However, in the elderly (65–75 years), the second most consumed group were fruits, followed by dairy products (Table 3). It is important to highlight that the consumption of cereals and derivatives, milk and dairy products, meats and meat products, sugars and sweets, appetizers, ready-to-eat meals, and sauces and condiments were significantly higher in children than the intakes by the adult and older adult populations (*p* ≤ 0.05). In particular, milk and dairy products intake were significantly higher in children compared to adolescents (*p* ≤ 0.05). On the contrary, intakes of vegetables, fruits, oils and fats, fish and shellfish, and alcoholic beverages were significantly higher in adults and the elderly (*p* ≤ 0.05). Similarly, it should be noted that, although the vegetable consumption was higher in adults and older adults when compared to the infant–juvenile population, older adults were the only group with a significantly higher consumption of this food group (*p* ≤ 0.05). However, adults between 18 and 64 years old consumed the highest quantities of drinks (*p* ≤ 0.05); both alcoholic and non-alcoholic (Table 3).

Male children and adolescents consumed higher levels of meats and processed meats, milk and derivatives, sugar and sweets, ready-to-eat meals, and sauces and condiments than adult groups (*p* < 0.05) (Table 4). In addition, children, adolescents, and adults showed a higher meat and derivative consumption than older adults (*p* < 0.05). Conversely, adults and elderly male populations presented a higher intake of vegetables, fruits, fats and oils, and fish and shellfish than children and adolescents (*p* < 0.05). Lastly, it is noteworthy that adults had a higher consumption of beverages, non-alcoholic as well as alcoholic (*p* < 0.05).

Children and adolescent females consumed more cereals and derivatives, meat and meat products, sugars and sweets, prepared meals, and sauces and condiments (*p* < 0.05) (Table 5). Likewise, it should be noted that girls consumed more milk and derivatives than the rest of the other population groups (*p* < 0.05). On the contrary, adult or elderly women had a higher consumption of vegetables, fruits, fish and shellfish and alcoholic beverages (*p* < 0.05). In addition, adult females had the highest consumption of non-alcoholic beverages.

## 4. Discussion

Dietary habits and food consumption patterns from the population are continuously changing and linked to the evolution of lifestyles and food availability. It is important to emphasize that there has been a rapid evolution in food patterns and, in the last decades, this has been reflected in the mortality and morbidity patterns amongst developed countries, which in turn translates them into a major public health problem, Spain being no exception [14]. At present, it is widely acknowledged that a suboptimal diet is an important preventable risk factor for NCDs. In fact, the Global Burden of Disease collaborators estimated in 2017 that low intake of whole grains (3 million deaths [2,3,4]) and low intake of fruits (2 million deaths [1,2,3,4]) were amongst the leading dietary risk factors for deaths worldwide [10]. In addition, it has been projected that by 2030, approximately 69% of all deaths worldwide will be attributable to NCDs [15]. The influence of dietary intakes as a modifiable risk factor has therefore raised a great interest for food consumption studies, as they represent the first step toward developing feasible prevention strategies [10]. Specifically, over the last three decades, food, work, and home environments have experienced numerous and determinant changes that influenced dietary patterns and food consumption amongst Spanish society [16,17]. Overweight and/or obesity are currently affecting more than 50% of adults and 25% of the infant/young population, thus it seems clear that these changes in dietary patterns and lifestyles will have negative consequences for both the present and future generations [18]. Therefore, we have analyzed and described the current food patterns from the Spanish ANIBES study population, being conscious of the difficulties in achieving accurate knowledge of dietary intakes.

The concept of the “Mediterranean Diet” emerged in the 50s with the aim of teaching the population about the dietary patterns and lifestyle of the countries surrounding the Mediterranean Sea [19] which, historically, have been associated with good health and quality of life [8]. The Mediterranean diet is characterized by the abundance of vegetable products (fruits, vegetables, cereals—especially whole grains—nuts, and legumes); the consumption of olive oil as the main source of dietary fat; a moderate intake of wine and other fermented beverages in meals; a frequent consumption of fresh fish, a moderate intake of dairy products (e.g., yogurt and certain cheeses); white meats; and eggs and low consumption in frequency and quantity of red meats and meat products (sausages and processed products) [8,20,21]. However, although Spain is bathed by the Mediterranean Sea, several studies indicate that over the last decades, the adherence of the Spanish population to this food pattern has decreased [16,22,23,24,25,26,27]. Particularly, the non-alcoholic beverages group has greatly evolved towards increased consumption, and a trend also greatly impacting on the global diet. A systematic review including 65 studies of beverage consumption, conducted between 2000 and 2013 and across different age groups, reported that total beverage intake was in the range of 0.6–1.8 L per day for children, 0.8–2.0 L per day for teenagers, and 0.8–3.4 L per day for adults, and water consumption accounted for 58%, 75%, and 80% of total fluid intake, respectively [28]. Moreover, a recent cross-national analysis of 75 countries has shown that sweetened soft drink consumption globally has increased from 36 L per person per year in 1997 to 43 L in 2010 [29]. In Spain, the evolution of non-alcoholic beverage consumption has experienced a great growth in recent decades, which has led to an increase of 27% in the consumption of non-alcoholic beverages per day from 2000 to the year 2008 [22]. Conversely, a moderate decrease in alcoholic beverages consumption is being observed in the Spanish adult population over the last decades, although present intake should be still considered a public health concern, especially for men. In addition, a shift toward a higher beer consumption instead of the more traditional Mediterranean wine has recently occurred [22]. Other studies mainly emphasize the contribution of beverages to energy and nutrient intakes [30,31] and how the increased beverage consumption has affected diet quality, namely, the caloric profile. Beverage consumption is a major contributor to daily energy intake, but it is often overlooked by individuals as a part of their dietary intakes [32]. Indeed, the types of drinks and their frequency of consumption might have either a negative or a positive impact in our overall nutrient intake. At present, the average energy consumption of the Spanish population in the ANIBES study is 1810 kcal/day, of which 12% are delivered by beverages. Water was the most consumed beverage, followed by milk, and the contribution of caloric soft drinks to the energy intake was 2% [13]. However, other population groups from France or Italy show a lower proportion of energy provided by beverages (8% and 6%, respectively) [33,34]. These are very close to the quantities suggested by international authorities who recommended that no more than 10% of the daily calorie intakes should come from beverages other than water [35,36].

The second most consumed group by the population of the ANIBES study are milk and dairy products. Still, despite this fact, it has experienced a drastic reduction in recent years. In the year 2000, consumption was 416 g/day [22] while the current consumption is 257 g/day, which shows a reduction by almost half. The data profile is pretty similar to those obtained from the Household Consumption Survey (Ministry of Agriculture, Fisheries and Food) [37] which shows a continuous fall in the consumption of dairy products, mainly for fluid milk, as their consumption in Spanish households in the year 2010 was 5246 tons versus 4937 tons in the year 2017. In this regard, it is relevant to underline that proteins from milk products are of high biological value and that they also provide other nutrients with beneficial effects on certain chronic diseases (fats, carbohydrates, and vitamins and minerals such as calcium and phosphorus) [38]. Furthermore, when we compare milk and dairy product intakes with other European countries, we can observe that populations from the Netherlands has a higher consumption (about 319 g/day, excluding cheese) than the population in Spain (257.2 g/day) and Belgium (160 g/day) [39,40].

Consumption of vegetables and greens, including potatoes, remained largely unchanged from the year 2000 (300 g/d) to 2008 (327 g/d) and fruits from the year 2000 (278 g/day) to 2008 (305 g/day) [22]. In turn, vegetable and fruit consumption amongst the ANIBES population shows a reduction to 178 g/day and 158 g/day, respectively. These data greatly differ from dietary recommendations for the Spanish population, specifically, those by the Spanish Society of Community Nutrition (SENC) [41] that endorse a minimum consumption of vegetables of at least 300 g/day (2 servings) and up to 400 g/day (3 servings) of fruits. These recommendations are based on the strong evidence that relates this food group with cardiovascular disease protection; possible evidence for decreased risk of colon cancer, pancreatic diseases, hip fracture, stroke, depression, and pancreatic diseases [42]. When assessing the number of servings of fruits and vegetables across the Spanish population, a report from the Spanish Nutrition Foundation (FEN) showed that the Spanish population is consuming roughly above 2 servings/day versus the recommended 5 servings/day [43]. When compared with other European countries we may observe that vegetable and fruit intakes in Dutch [39] and Belgian [40] populations are similar but lower than in Italy where vegetable consumption reaches 244 g/day and fruit 239.6 g/day [44].

Historically, carbohydrate intakes within the Spanish diet came mainly from grain and derivatives, with bread as the main contributor [5]. In Spain, the evolution of global consumption of the cereals and derivatives group has undergone only a small variation from year 2000 to present [22]. However, if we compare this consumption data with previous decades, a marked decrease in consumption occurred, since in 1964 it was 436 g/person per day in Spanish households, according to the “Family Budget Survey” carried out by the National Institute of Statistics in 1991 [24]. Moreover, the European average in terms of annual consumption of cereals is higher than the Spanish average, around 2.7 kilos per person, with Ireland, Sweden, and Finland being the countries with the highest consumption (above 7 kg per person) [45]. This decrease in the consumption of cereals and derivatives may be due to the rapid evolution of dietary habits along with the abandonment of “basic foods” replaced by other groups that are more processed and transformed [46]. In addition, the lower consumption of bread in our country has been related to a misperception that it might increase weight, but also a lower quality of bread.

It is also worth highlighting that in Spain there has been a moderate decrease in the consumption of meat and derivatives in the past few years. In line with data from the Food Panel in the year 2000 [22], meat and derivatives intakes were close to 180 g/day while, at present, results from the ANIBES study show that average daily consumption is near to 146 g/day, and they are the main protein sources for the whole population (33.1%) [5]. Nevertheless, mean intake of red meat by the ANIBES population was still higher than the maximum of 3 weekly servings which are being recommended by the SENC Dietary Guidelines [41]. The latter should be also considered within the latest guidelines published by the World Health Organization (WHO), suggesting to avoid high intakes associated with potential deleterious health effects [47]. Moreover, these intakes unquestionably must be considered as high according to the traditional Mediterranean dietary patterns. Other relevant protein sources are fish and shellfish, as an important number of studies have underlined the role of fish intake in the primary and secondary prevention of cardiovascular disease, mainly because of their high content in long-chain n-3 polyunsaturated fatty acids [48,49]. Fish and shellfish are also a good source of key nutrients such as vitamin D, A, and calcium [50,51]. However, their consumption has decreased approximately 30% in the past few years in Spain and mainly for the youngest; in the year 2000, average intakes amongst the Spanish population were 88.9 g/day [22] and at present are about 62.0 g/day per person, which, in any case, represent a higher consumption than the observed amongst the Italian population (33.7 g/day) [44] and, still, Spain retains the second highest world consumption.

Likewise, eggs, which are an optimal source of unsaturated fats, high-quality protein, folates, and many vitamins and minerals [52], have experienced a decrease from observed intakes in the year 2000, which were 4.3 medium-sized eggs per week [22,25]. Probably, this was a consequence of bad press received health wise, associated to their high cholesterol content. Our results showed egg intakes of 29.3 g/day, significantly higher in men (32.8 g/day), which is in agreement with previous results from the Food Consumption Survey—32 g/day [25]—still low compared to intakes from the year 2000. Nonetheless, the latest Dietary Guidelines for the Spanish population [41] feature that the consumption of 3 to 5 egg units per week may represent a good nutritional alternative to meat and fish products. This recommendation encourages the increase in egg consumption as a nutritious food source.

Oils and fats represented the main sources of lipids for the Spanish population of which, fortunately, olive oil accounted for 36.9% of the monounsaturated fatty acid intakes [5]. The consumption of oils and fats by the ANIBES population was 25 g/day, which shows a considerable reduction in this group intake, since in the year 2000 their consumption was 49.2 g/day. Fats and oils intakes amongst the Dutch and Belgian populations are quite comparable to the consumption by the Spanish population but is lower than the one observed amongst Italians (33.1 g/day) [44]. It is worth underlining that the consumption of olive oil by the ANIBES population was 17.5 g/day, which reflects that although the total intake of this food group has been reduced, there is a predilection for this culinary fat as a key dietary component of Mediterranean countries. Olive oil is at the core of the Mediterranean diet and it is the best fat source, as it has been recognized for centuries for its nutritional quality and health associated effects [39,53]. Other fat sources, such as sunflower and vegetable-refined oils, represent a minor percentage of the ANIBES population consumption (4.4 ± 5.2 g/day). Moreover, butter and margarine consumption were even lower (3.5 ± 6.2 g/day) when compared with intakes from other countries such as the United Kingdom and Germany [54,55]. The latter might be explained in part as these are not mainly used as cooking fats or added to food dishes as salads, where Spain still prioritizes olive oil—in fact, the Mediterranean food legacy.

Legumes and pulses are one of the food groups for which intakes have negatively decreased in the last decades in Spain. Specifically, in the year 1964, their average consumption was about 41 g/day [27] and at present it is only 13 g/day. One goal of the Spanish SENC guidelines [41] is to increase pulse consumption by encouraging people to eat a 60 to 80 g serving at least two to four times per week (versus roughly 1 serving size per week at present), since these foods are a primary source of protein, fiber, and other essential nutrients that may have a potential role in colon cancer prevention and reduction of cholesterol levels, as well as being a key element for a better adherence to the Mediterranean dietary pattern.

Interestingly, our analysis demonstrates important differences in food consumption patterns between men and women. Men consumed more cereals and derivatives, meat and meat products, eggs, ready-to-eat meals, sauces and condiments, and alcoholic beverages than women did, whereas women consumed more fruits. These findings are generally consistent with findings from other studies [56,57,58]. A possible explanation for this might be that women are frequently more weight-conscious and believe that it may be advisable to limit carbohydrates and fat consumption [59]. In addition, they usually have healthier eating habits than men [60]. Likewise, age has a strong influence in food selection, as our results show that children and young individuals consume a higher proportion of cereals and derivatives, milk and dairy products, sugar and sweets, and ready-to-eat meals, while adults have higher vegetable, fruit, and fish and shellfish intakes. Several studies support these findings, showing that consumption of fruit and vegetables generally increase during the transition of the population from a young to adult age, while intakes of cereals and milk and dairy products tend to decrease [40]. In the United Kingdom, Lake et al. [61] reported that food group intakes changed considerably between adolescence and adulthood. In this longitudinal study conducted from 1980 to 2000, with those between the ages of 12 and 33 y, consumption of foods containing fat and/or sugar and milk and dairy foods decreased, while intakes of fruits and vegetables increased. Intakes of bread, other cereals and potatoes; fruits and vegetables and meat; and fish and alternatives remained constant from adolescence to adulthood. The authors noted that although adolescent eating patterns are commonly described as unhealthy, in their study sample, some dietary patterns were worth maintaining to achieve a healthy diet at an older age. These facts underline the importance of acquiring and educating healthy eating patterns, culinary skills, and gastronomic experiences in childhood and adolescence ages.

### Strengths and Limitations of the Present Study

The strengths of the study include the careful design, protocol, and methodology; the use of a representative sample of the Spanish population aged 9 to 75 years; the innovative methodology for compiling dietary data and dietary assessment method; as well as the extended and updated food database created that allowed to obtain data not only from major food groups but also subgroups of key interest. A limitation of this study is its cross-sectional design, which provides evidence for associations but not for causal relationships. Another type of limitation to consider is what food patterns were established based on a 3 day meal record and whether it is possible that the foods occasionally consumed were not properly valued.

## 5. Conclusions

The present study showed that the majority of the Spanish population, mainly the younger groups, is moving away from the traditional Mediterranean dietary pattern, compared to a few decades ago. The results obtained according to different ages, gender, and for different food groups and subgroups, reinforce the idea that tailored food and nutrition policies are urgently needed.

## Figures and Tables

**Table 1:** Statistical description of the(ANIBES) “anthropometric data, macronutrients and micronutrients intake, practice of physical activity, socioeconomic data, and lifestyles in Spain” study population sample (Modified from Nissensohn et al. [13]).

		Total	%	Male	%	Female	%
		2007	100	1011	50.4	996	49.6
Age Group	Children (9–12 years)	100	5.0	62	6.1	38	3.8
	Adolescents (13–17 years)	123	6.1	84	8.3	39	3.9
	Adults (18–64 years)	1587	79.1	772	76.4	815	81.9
	Older adults (65–75 years)	197	9.8	93	9.2	104	10.4
Level of education	Primary or less	743	37	378	37.4	365	36.6
	Secondary	858	42.8	434	42.9	424	42.6
	Tertiary or University	406	20.2	199	19.7	207	20.8
Economical level	1000 € or less	397	19.8	191	18.9	206	20.7
	From 1000 to 2000 €	795	39.6	393	38.9	402	40.4
	Over 2000 €	320	15.9	163	16.1	157	15.8
	No income	7	0.3	4	0.4	3	0.3
	No answer	488	24.3	260	25.7	228	22.9
Geographical distribution	Northwest	152	7.6	77	7.6	75	7.5
	North Central	161	8	79	7.8	82	8.2

**Table 2:** Daily food groups and subgroups intake distribution amongst the Spanish ANIBES study population (9–75 years).

Food Groups and Subgroups	Total Population	Men	Women
Cereals and derivatives (g/day)	148.9 ± 63.8	163.8 ± 67.9	133.8 ± 55.4 ***
Grains and flours (g/day)	22.5 ± 26.4	23.8 ± 28.6	21.2 ± 23.9 *
Breakfast cereals and cereal bars (g/day)	4.7 ± 12.9	5.0 ± 14.9	4.4 ± 10.5
Bread (g/day)	75.0 ± 45.2	84.9 ± 49.6	64.9 ± 37.8 ***
Pasta(g/day)	16.9 ± 20.9	19.2 ± 23.1	14.6 ± 18.1 ***
Bakery and pastry (g/day)	29.8 ± 33.9	30.9 ± 35.7	28.8 ± 31.9
Vegetables (g/day)	177.8 ± 112.9	175.3 ± 116.0	180.3 ± 109.6
Fruits(g/day)	158.0 ± 175.4	149.6 ± 173.9	166.5 ± 176.6 *
Oils and fats (g/day)	24.5 ± 11.2	24.8 ± 11.5	24.3 ± 10.8
Olive oil (g/day)	17.5 ± 9.4	17.8 ± 9.8	17.1 ± 9.0
Other oils (g/day)	3.6 ± 5.1	3.9 ± 5.6	3.3 ± 4.6 **
Butter. margarine and shortening (g/day)	3.5 ± 7.0	3.1 ± 7.0	3.9 ± 7.1 **
Milk and dairy products (g/day)	257.2 ± 159.5	258.4 ± 170.5	256.1 ± 146.6
Milks (g/day)	173.4 ± 132.8	171.1 ± 139.0	175.8 ± 126.3
Cheeses (g/day)	18.0 ± 23.0	18.4 ± 66.7	17.6 ± 60.7
Yogurt and fermented milk (g/day)	46.5 ± 63.7	46.2 ± 23.9	46.8 ± 19.7
Other dairy products (g/day)	19.4 ± 44.1	22.8 ± 50.6	16.0 ± 35.8 ***
Fish and shellfish (g/day)	62.3 ± 69.9	60.8 ± 68.2	63.9 ± 71.6
Meat and meat products (g/day)	146.0 ± 82.8	165.4 ± 87.8	126.3 ± 72.2 ***
Meat (g/day)	103.9 ± 73.8	117.4 ± 78.8	90.3 ± 65.6 ***
Viscera and spoils (g/day)	40.5 ± 36.4	46.1 ± 9.6	34.7 ± 7.6
Sausages and other meat products (g/day)	1.6 ± 8.7	1.9 ± 39.6	1.3 ± 31.8 ***
Eggs (g/day)	29.3 ± 29.4	32.8 ± 32.9	25.7 ± 25.0 ***
Pulses (g/day)	13.5 ± 17.8	14.1 ± 18.8	12.8 ± 16.8
Sugar and sweets (g/day)	15.9 ± 16.0	15.4 ± 16.1	16.4 ± 16.0
Sugar (g/day)	6.2 ± 8.4	6.0 ± 8.2	6.5 ± 8.5
Chocolates (g/day)	6.8 ± 11.4	7.2 ± 11.9	6.4 ± 10.9
Jams and other (g/day)	2.2 ± 6.4	1.7 ± 5.7	2.7 ± 7.1 ***
Other sweets (g/day)	0.7 ± 3.7	0.5 ± 3.2	0.9 ± 4.2
Appetizers (g/day)	5.5 ± 12.3	5.9 ± 12.6	5.1 ± 1.9
Ready-to-eat-meals (g/day)	70.2 ± 77.6	79.4 ± 86.2	60.9 ± 66.5 ***
Sauces and condiments (g/day)	12.9 ± 14.5	14.4 ± 15.3	11.4 ± 13.5 ***
Non-alcoholic Beverages (g/day)	850.8 ± 521.3	842.1 ± 540.6	859.7 ± 501.1
Water (g/day)	569.7 ± 502.3	557.7 ± 517.9	581.9 ± 485.8
Coffee and infusions (g/day)	86.2 ± 117.9	75.0 ± 109.2	97.6 ± 125.2 ***
Sugary soft drinks (g/day)	88.2 ± 151.3	103.2 ± 170.9	73.0 ± 126.5 ***
Non-sweetened soft drinks (g/day)	35.4 ± 108.8	29.2 ± 91.9	41.6 ± 123.3 **
Sports Drinks (g/day)	4.9 ± 33.9	6.8 ± 41.3	2.9 ± 24.1 **
Juices and nectars (g/day)	50.6 ± 94.4	56.3 ± 104.0	44.9 ± 83.2 **
Other drinks (non-alcoholic) (g/day)	14.2 ± 55.6	11.5 ± 55.0	16.9 ± 56.1 *
Energy drinks (g/day)	1.7 ± 20.6	2.4 ± 26.8	1.0 ± 11.2
Alcoholic Beverages (g/day)	99.2 ± 195.8	135.4 ± 238.0	62.4 ± 130.8 ***
High alcohol content (g/day)	2.2 ± 13.5	2.8 ± 15.1	1.5 ± 11.6 **
Low alcohol content (g/day)	97.0 ± 192.9	132.6 ± 234.0	60.9 ± 129.5 ***
Supplements and meal replacements (g/day)	0.4 ± 4.6	0.5 ± 5.1	0.3 ± 4.0

Data reported as means ± standard deviation (SD) per group. * *p* < 0.05 difference male (Student’s *t*-test); ** *p* < 0.01 difference male (Student’s t-test); *** *p* < 0.001 difference male (Student’s *t*-test).

**Table 3:** Total daily food groups and subgroups intake by age amongst the Spanish ANIBES study population.

Food Groups and Subgroups	Children (9–12 years)	Adolescents (13–17 years)	Adults (18–64 years)	Older Adults (65–75 years)
Cereals and derivatives (g/day)	168.4 ± 58.6 ^a,b^	180.8 ± 71.9 ^a^	148.8 ± 64.4 ^b^	126.3 ± 53.2 ^c^
Grains and flours (g/day)	21.1 ± 23.0	25.1 ± 27.1	22.8 ± 26.9	17.6 ± 22.0
Breakfast cereals and cereal bars (g/day)	7.8 ± 15.7	9.7 ± 18.5	4.4 ± 12.1	3.0 ± 9.7
Bread (g/day)	78.4 ± 44.7	83.7 ± 52.5	75.0 ± 45.4	70.9 ± 44.3
Pasta(g/day)	20.3 ± 17.5	22.8 ± 25.1	17.2 ± 21.3	10.0 ± 14.0
Bakery and pastry (g/day)	40.8 ± 32.4	39.5 ± 39.9	29.4 ± 33.7	24.9 ± 29.6
Vegetables (g/day)	124.8 ± 92.9 ^d^	122.2 ± 85.0 ^d^	181.1 ± 115.3 ^e^	210.3 ± 110.4 ^f^
Fruits(g/d)	104.9 ± 104.1 ^g^	87.0 ± 114.7 ^g^	151.5 ± 170.4 ^h^	280.8 ± 222.0 ^i^
Oils and fats (g/day)	23.0 ± 9.5 ^j,k^	22.2 ± 12.3 ^j^	24.6 ± 11.1 ^j,k^	26.8 ± 13.2 ^k^
Olive oil (g/day)	15.1 ± 7.9	14.3 ± 9.2	17.4 ± 9.3	21.0 ± 9.9
Other oils (g/day)	4.4 ± 5.2	4.5 ± 7.1	3.7 ± 5.2	1.4 ± 2.9
Butter, margarine, and shortening (g/day)	3.5 ± 6.2	3.5 ± 7.9	3.4 ± 6.6	4.3 ± 10.6
Milk and dairy products (g/day)	360.1 ± 149.6 ^l^	307.0 ± 188.3 ^m^	247.6 ± 154.8 ^n^	260.4 ± 159.7 ^n^
Milks (g/day)	232.7 ± 132.0	216.5 ± 134.9	165.8 ± 129.9	184.6 ± 140.3
Cheeses (g/day)	18.5 ± 64.7	18.7 ± 64.6	18.5 ± 62.2	12.7 ± 70.7
Yogurt and fermented milk (g/day)	58.3 ± 16.2	39.1 ± 18.2	44.9 ± 22.9	54.9 ± 16.7
Other dairy products (g/day)	50.6 ± 76.7	32.8 ± 61.6	18.4 ± 40.5	8.2 ± 20.8
Fish and shellfish (g/day)	45.4 ± 54.6 ^m^	40.2 ± 57.9 ^m^	62.6 ± 70.4 ^o^	81.1 ± 72.5 ^p^
Meat and meat products (g/day)	148.4 ± 69.5 ^q^	158.8 ± 71.4 ^q^	148.4 ± 84.4 ^q^	117.0 ± 70.0 ^r^
Meat (g/day)	97.1 ± 61.8	109.4 ± 69.8	106.2 ± 75.6	85.9 ± 61.3
Viscera and spoils (g/day)	51.0 ± 3.2	48.9 ± 5.1	40.7 ± 8.3	27.9 ± 13.2
Sausages and other meat products (g/day)	0.3 ± 39.0	0.5 ± 36.9	1.5 ± 36.4	3.2 ± 28.9
Eggs (g/day)	27.0 ± 24.4	31.3 ± 34.1	29.3 ± 29.5	32.4 ± 29.2
Pulses (g/day)	12.2 ± 14.3	12.1 ± 16.2	13.4 ± 18.0	15.2 ± 18.6
Sugar and sweets (g/day)	24.6 ± 19.9 ^s^	21.9 ± 18.7 ^s^	15.6 ± 15.5 ^t^	12.3 ± 13.7 ^u^
Sugar (g/day)	2.6 ± 5.2	4.0 ± 7.4	6.7 ± 8.7	5.4 ± 7.6
Chocolates (g/day)	19.1 ± 17.3	15.5 ± 15.1	6.1 ± 10.1	2.0 ± 5.3
Jams and other (g/day)	1.3 ± 4.0	1.1 ± 4.0	2.1 ± 6.2	4.7 ± 10.2
Other sweets (g/day)	1.5 ± 5.5	1.3 ± 5.6	0.7 ± 3.7	0.2 ± 0.7
Appetizers (g/day)	5.1 ± 10.1 ^v,w^	5.7 ± 12.5 ^v,w^	5.8 ± 12.7 ^v^	2.9 ± 9.0 ^w^
Ready-to-eat meals (g/day)	83.0 ± 75.3 ^x^	95.8 ± 93.4 ^x^	67.5 ± 75.7 ^y^	60.3 ± 68.3 ^y^
Sauces and condiments (g/day)	15.9 ± 17.0 ^z^	15.4 ± 15.0 ^z^	13.3 ± 14.8 ^z^	6.8 ± 10.2 ^α^
Non-alcoholic beverages (g/day)	707.8 ± 408.8 ^β^	699.4 ± 381.3 ^β^	885.1 ± 535.5 ^Ω^	700.5 ± 435.2 ^β^
Water (g/day)	481.4 ± 387.3	407.1 ± 358.4	591.3 ± 519.8	496.9 ± 414.7
Coffee and infusions (g/day)	4.1 ± 18.2	11.2 ± 37.9	93.9 ± 115.1	112.2 ± 159.1
Sugar soft drinks (g/day)	84.4 ± 139.0	150.6 ± 190.0	89.0 ± 149.8	23.8 ± 63.6
Non-sweetened soft drinks (g/day)	15.1 ± 46.8	20.5 ± 76.2	41.0 ± 118.6	13.1 ± 50.8
Sports drinks (g/day)	9.5 ± 51.0	2.0 ± 16.5	6.0 ± 40.6	1.0 ± 10.4
Juices and nectars (g/day)	111.7 ± 137.8	99.7 ± 147.7	46.6 ± 86.4	33.0 ± 78.1
Other drinks (non-alcoholic) (g/day)	1.2 ± 17.1	4.4 ± 28.9	15.9 ± 58.8	20.5 ± 69.4
Energy drinks (g/day)	0.5 ± 7.5	3.9 ± 29.1	1.5 ± 20.1	0.0 ± 0.0
Alcoholic beverages (g/day)	0.0 ± 0.0 ^π^	1.8 ± 16.0 ^π^	113.4 ± 209.1 ^µ^	96.4 ± 156.3 ^µ^
High alcohol content beverage (g/day)	0.0 ± 0.0	0.0 ± 0.0	2.5 ± 14.3	2.1 ± 14.5
Low alcohol content beverage (g/day)	0.0 ± 0.0	1.8 ± 16.0	110.8 ± 206.1	94.3 ± 153.7
Supplements and meal replacements (g/day)	0.0 ± 0.2	0.1 ± 1.5	0.4 ± 5.0	0.6 ± 5.7

Data reported as means ± standard deviation (SD) per group. Different superscript lowercase letters indicate statistical significance in each row for food groups (*p* ≤ 0.05; Bonferroni test).

**Table 4:** Total daily food groups and subgroups intake by age in the male Spanish ANIBES study population.

Food Groups and Subgroups	Children	Adolescents	Adults	Older Adults
	(9–12 years)	(13–17 years)	(18–64 years)	(65–75 years)
Cereals and derivatives (g/day)	169.9 ± 60.2 ^a^	192.6 ± 74.6 ^b^	163.7 ± 68.5 ^a^	137.2 ± 59.0 ^c^
Grains and flours (g/day)	20.9 ± 20.3	25.7 ± 28.3	24.2 ± 29.5	20.1 ± 25.2
Breakfast cereals and cereal bars (g/day)	8.2 ± 17.9	11.4 ± 21.2	4.3 ± 13.5	3.1 ± 8.9
Bread (g/day)	80.2 ± 45.9	88.9 ± 54.6	85.5 ± 50.1	79.1 ± 47.7
Pasta(g/day)	20.3 ± 16.8	24.9 ± 26.1	19.9 ± 23.9	9.2 ± 13.0
Bakery and pastry (g/day)	40.4 ± 32.8	41.8 ± 44.8	29.8 ± 34.9	25.8 ± 32.6
Vegetables (g/day)	120.6 ± 78.0 ^d^	123.7 ± 90.0 ^d^	181.0 ± 118.7 ^e^	214.7 ± 108.0 ^f^
Fruits(g/day)	96.7 ± 96.2 ^g^	82.0 ± 116.9 ^g^	147.5 ± 167.8 ^h^	279.0 ± 244.9 ^i^
Oils and fats (g/day)	23.1 ± 9.9 ^j^	23.0 ±12.7 ^j^	24.8 ± 11.3 ^j,k^	27.7 ± 13.2 ^k^
Olive oil (g/day)	15.3 ± 8.3	15.1 ± 10.0	17.8 ± 9.8	22.4 ± 9.8
Other oils (g/day)	4.2 ± 4.8	4.4 ± 6.8	4.1 ± 5.6	1.6 ± 3.3
Butter, margarine and shortening (g/day)	3.6 ± 6.5	3.5 ± 8.5	2.9 ± 6.1	3.7 ± 10.8
Milk and dairy products (g/day)	384.2 ± 148.7 ^l^	331.6 ± 200.0 ^l^	246.5 ± 167.0 ^m^	242.3 ± 159.6 ^m^
Milks (g/day)	252.2 ± 137.0	241.0 ± 177.2	160.2 ± 136.5	176.9 ± 145.1
Cheeses (g/day)	20.0 ± 23.3	18.3 ± 22.7	19.1 ± 26.1	10.7 ± 14.8
Yogurt and fermented milk (g/day)	57.9 ± 62.0	36.6 ± 54.8	46.2 ± 68.3	47.7 ± 67.5
Other dairy products (g/day)	54.1 ± 80.0	35.7 ± 67.8	20.9 ± 45.3	7.0 ± 17.9
Fish and shellfish (g/day)	45.7 ± 59.4 ^n^	40.2 ± 55.3 ^n^	61.4 ± 68.6 ^o^	90.3 ± 76.7 ^p^
Meat and meat products (g/day)	159.3 ± 73.0 ^q,r^	171.4 ± 66.0 ^r^	169.2 ± 73.6 ^r^	132.5 ± 76.0 ^q^
Meat (g/day)	107.7 ± 64.5	120.2 ± 70.1	120.7 ± 81.2	95.5 ± 66.4
Sausages and other meat products (g/day)	51.6 ± 1.5	50.9 ± 2.8	46.6 ± 9.7	33.1 ± 13.6
Viscera and spoils (g/day)	0.1 ± 1.5	0.2 ± 2.8	1.9 ± 9.7	3.9 ± 13.6
Eggs (g/day)	27.7 ± 25.6	34.7 ± 36.1	33.2 ± 33.4	34.5 ± 29.5
Pulses (g/day)	11.0 ± 14.8	12.0 ± 15.3	14.3 ± 19.2	17.3 ± 20.0
Sugar and sweets (g/day)	24.4 ± 21.0 ^s^	20.8 ± 16.4 ^s^	14.8 ± 15.5 ^t^	12.7 ± 14.0 ^t^
Sugar (g/day)	2.6 ± 5.1	4.3 ± 8.1	6.6 ± 8.8	5.6 ± 7.4
Chocolates (g/day)	19.2 ± 17.5	14.6 ± 13.8	6.2 ± 10.6	3.0 ± 6.4
Jams and other (g/day)	1.1 ± 3.6	1.0 ± 3.7	1.6 ± 5.4	4.0 ± 9.5
Other sweets (g/day)	1.4 ± 6.4	0.9 ± 3.5	0.5 ± 2.9	0.2 ± 0.7
Appetizers (g/day)	5.0 ± 9.8	5.4 ± 12.4	6.4 ± 13.3	3.3 ± 8.4
Ready-to-eat meals (g/day)	88.9 ± 72.9 ^u,v^	107.5 ± 101.2 ^u^	74.1 ± 84.1 ^v^	76.3 ± 74.5 ^v^
Sauces and condiments (g/day)	14.4 ± 12.9 ^x^	16.0 ± 13.9 ^x^	15.0 ± 15.9 ^x^	7.5 ± 11.6 ^y^
Non-alcoholic beverages (g/day)	728.4 ± 435.2 ^z^	714.0 ± 418.4 ^z^	884.2 ± 561.4 ^α^	640.8 ± 429.4 ^Z^
Water (g/day)	487.6 ± 402.6	416.8 ± 387.3	584.1 ± 541.8	452.7 ± 403.1
Coffee and infusions (g/day)	2.6 ± 13.0	12.8 ± 43.4	86.0 ± 114.1	88.7 ± 106.7
Sugar soft drinks (g/day)	99.1 ± 158.2	158.6 ± 215.3	102.5 ± 167.2	24.6 ± 63.7
Non-sweetened soft drinks (g/day)	12.0 ± 35.3	21.5 ± 74.4	34.8 ± 103.5	8.0 ± 31.2
Sports drinks (g/day)	13.0 ± 61.9	1.3 ± 11.0	8.8 ± 51.9	1.3 ± 13.4
Juices and nectars (g/day)	112.0 ± 146.8	94.8 ± 137.6	53.3 ± 100.8	34.3 ± 79.6
Other drinks (non-alcoholic) (g/day)	2.0 ± 22.3	2.0 ± 12.4	12.6 ± 58.0	31.3 ± 87.0
Energy drinks (g/day)	0.0 ± 0.0	6.1 ± 35.9	2.1 ± 26.4	0.0 ± 0.0
Alcoholic beverages (g/day)	0.0 ± 0.0 ^β^	1.1 ± 1.1 ^β^	159.3 ± 9.0 ^µ^	143.5 ± 19.0 ^µ^
High alcohol content beverage (g/day)	0.0 ± 0.0	0.0 ± 0.0	3.2 ± 15.6	4.4 ± 20.8
Low alcohol content beverage (g/day)	0.0 ± 0.0	1.1 ± 13.2	156.0 ± 251.0	139.1 ± 185.6
Supplements and meal replacements (g/day)	0.0 ± 0.3	0.2 ± 1.9	0.5 ± 5.7	1.1 ± 8.2

Data reported as means ± standard deviation (SD) per group. Different superscript lowercase letters indicate statistical significance in each row for food groups (*p* ≤ 0.05; Bonferroni test).

**Table 5:** Total daily food groups and subgroups intake by age in the female Spanish ANIBES study population.

Food Groups and Subgroups	Children	Adolescents	Adults	Older Adults
	(9–12 years)	(13–17 years)	(18–64 years)	(65–75 years)
Cereals and derivatives (g/day)	166.3 ± 58.5 ^a^	158.9 ± 63.2 ^a^	134.9 ± 56.4 ^b^	116.2 ± 45.5 ^b^
Grains and flours (g/day)	21.4 ± 26.5	23.8 ± 24.8	21.5 ± 24.2	15.3 ± 18.4
Breakfast cereals and cereal bars (g/day)	7.3 ± 11.9	6.7 ± 11.6	4.5 ± 10.6	2.9 ± 10.4
Bread (g/day)	75.8 ± 43.2	73.9 ± 47.2	65.1 ± 38.1	63.3 ± 39.6
Pasta(g/day)	20.4 ± 18.5	19.1 ± 22.7	14.7 ± 18.2	10.7 ± 14.9
Bakery and pastry (g/day)	41.4 ± 31.9	35.4 ± 28.5	29.0 ± 32.6	24.0 ± 29.2
Vegetables (g/day)	130.7 ± 111.2 ^c^	119.5 ± 75.4 ^c^	181.3 ± 112.2 ^d^	206.3 ± 113.0 ^d^
Fruits (g/day)	117.0 ± 114.1 ^e,f^	96.2 ± 110.5 ^e^	155.3 ± 172.7 ^f^	282.5 ± 199.6 ^g^
Oils and fats (g/day)	22.9 ± 8.8 ^h,i^	20.8 ± 11.6 ^h^	24.5 ± 10.9 ^i^	25.9 ± 13.3 ^i^
Olive oil (g/day)	14.8 ± 7.2	12.7 ± 7.2	17.1 ± 8.9	19.7 ± 9.8,
Other oils (g/day)	4.7 ± 5.7	4.6 ± 7.6	3.4 ± 4.6	1.3 ± 2.6
Butter, margarine and shortening (g/day)	3.4 ± 5.7	3.5 ± 6.7	3.9 ± 7.0	4.9 ± 10.5
Milk and dairy products (g/day)	325.1 ± 151.0 ^j^	261.4 ± 154.9 ^k^	248.6 ± 142.8 ^k^	277.2 ± 158.2 ^j,k^
Milks (g/day)	204.5 ± 119.6	171.1 ± 128.4	171.0 ± 123.3	191.8 ± 136.1
Cheeses (g/day)	16.3 ± 19.0	19.3 ± 19.8	17.9 ± 20.5	14.6 ± 19.0
Yogurt and fermented milk (g/day)	58.9 ± 68.7	43.5 ± 79.8	43.7 ± 55.9	61.6 ± 73.2
Other dairy products (g/day)	45.5 ± 71.7	27.5 ± 48.0	16.0 ± 35.3	9.3 ± 23.2
Fish and shellfish (g/day)	44.8 ± 47.2 ^l,m^	40.2 ± 62.8 ^m^	63.7 ± 72.1 ^l,n^	72.5 ± 67.7 ^n^
Meat and meat products (g/day)	132.5 ± 61.0 ^o^	135.7 ± 71.4 ^o^	129.0 ± 90.2 ^o^	102.7 ± 60.8 ^p^
Meat (g/day)	81.8 ± 54.5	89.5 ± 65.1	92.7 ±67.3	77.1 ± 55.0
Sausages and other meat products (g/day)	50.2 ± 4.6	45.2 ± 7.7	35.2 ± 6.8	23.1 ± 12.9
Viscera and spoils (g/day)	0.5 ± 4.6	0.9 ± 7.7	1.2 ± 6.8	2.5 ± 12.9
Eggs (g/day)	26.1 ± 22.5	25.0 ± 29.4	25.6 ± 24.9	30.4 ± 28.9
Pulses (g/day)	13.8 ± 13.5	12.2 ± 17.7	12.5 ± 16.8	13.3 ± 17.0
Sugar and sweets (g/day)	24.8 ± 18.2 ^q^	24.0 ± 22.4 ^q^	16.3 ± 15.5 ^r^	12.0 ± 13.4 ^s^
Sugar (g/day)	2.6 ± 5.4	3.4 ± 6.1	6.9 ± 8.7	5.2 ± 7.8
Chocolates (g/day)	19.0 ± 17.0	17.2 ± 17.1	6.1 ± 9.7	1.2 ± 4.0
Jams and other (g/day)	1.6 ± 4.5	1.4 ±4.4	2.5 ± 6.9	5.4 ± 10.8
Other sweets (g/day)	1.7 ± 4.0	2.1 ± 8.2	0.8 ± 4.3	0.2 ± 0.8
Appetizers (g/day)	5.2 ± 10.7	6.2 ± 12.9	5.3 ± 12.0	2.6 ± 9.6
Ready-to-eat meals (g/day)	74.4 ± 78.2 ^t^	74.1 ± 72.5 ^t,u^	61.4 ± 66.4 ^t,u^	45.5 ± 58.6 ^u^
Sauces and condiments (g/day)	17.9 ± 21.5 ^v^	14.3 ± 16.8 ^v^	11.8 ± 13.4 ^w^	6.1 ± 8.8 ^x^
Non-alcoholic beverages (g/day)	677.9 ± 367.6 ^y^	672.3 ± 301.6 ^y^	886.0 ± 510.6 ^z^	755.7 ± 435.2 ^y,z^
Water (g/day)	472.3 ± 366.3	389.1 ± 299.1	597.9 ± 498.6	537.7 ± 422.9
Coffee and infusions (g/day)	6.1 ± 23.6	8.1 ± 24.7	101.2 ± 115.7	133.9 ± 193.5
Sugar soft drinks (g/day)	63.0 ± 102.3	135.7 ± 130.8	76.4 ± 130.4	23.0 ± 63.8
Non-sweetened soft drinks (g/day)	19.6 ± 59.7	18.6 ± 79.9	46.7 ± 130.9	17.9 ± 63.7
Sports Drinks (g/day)	4.3 ± 28.3	3.3 ± 23.7	3.3 ± 25.7	0.6 ± 6.4
Juices and nectars (g/day)	111.2 ± 124.6	108.8 ± 165.4	40.4 ± 69.8	31.9 ± 77.0
Other drinks (non-alcoholic) (g/day)	0.0 ± 0.0	8.8 ± 45.7	19.0 ± 59.5	10.6 ± 46.1
Energy drinks (g/day)	1.3 ± 11.8	0.0 ± 0.0	1.0 ± 11.5	0.0 ± 0.0
Alcoholic beverages (g/day)	0.0 ± 0.0 ^α^	2.9 ± 2.3 ^α^	70.6 ± 4.8 ^β^	52.8 ± 9.8 ^β^
High alcohol content beverage (g/day)	0.0 ± 0.0	0.0 ± 0.0	1.9 ± 12.9	0.0 ± 0.0
Low alcohol content beverage (g/day)	0.0 ± 0.0	2.9 ± 20.2	68.8 ± 140.6	52.8 ± 101.1
Supplements and meal replacements (g/day)	0.0 ± 0.0	0.0 ± 0.0	0.3 ± 4.3	0.1 ± 0.4

Data reported as means ± standard deviation (SD) per group. Different superscript lowercase letters indicate statistical significance in each row for food groups (*p* ≤ 0.05; Bonferroni test).
